# Distinct roles of NK cells in viral immunity during different phases of acute Friend retrovirus infection

**DOI:** 10.1186/1742-4690-10-127

**Published:** 2013-11-01

**Authors:** Elisabeth Littwitz, Sandra Francois, Ulf Dittmer, Kathrin Gibbert

**Affiliations:** 1 Institute for Virology of the University Hospital in Essen, University of Duisburg-Essen, Essen, Germany

**Keywords:** NK cells, Friend retrovirus, Effector T cells, Regulatory function, Viral control

## Abstract

**Background:**

In many virus infections natural killer (NK) cells are critical for the rapid containment of virus replication. Polymorphisms in NK cell receptors as well as viral escape from NK cell responses are associated with pathogenesis and viral loads in HIV-infected individuals, emphasizing their importance in retroviral immunity. In contrast, NK cells of LCMV-infected mice dampened virus-specific T cell responses resulting in impaired virus control. Thus, the exact role of NK cells during different phases of viral infections remains elusive. In this study we characterized the NK cell response at different time points of an acute retroviral infection by using the Friend retrovirus (FV) mouse model.

**Findings:**

Depletion of NK1.1^+^ cells during the initial phase of FV infection (3 to 4 days post infection) resulted in increased viral loads, which correlated with enhanced target cell killing and elevated NK cell effector functions. At days 7 to 15 post infection, NK and NKT cells did not contribute to anti-retroviral immunity. In the transition phase between acute and chronic infection (30 days post infection), NK and NKT cells exhibited an inhibitory role and their depletion resulted in reduced viral loads and significantly improved FV-specific CD8^+^ T cell responses.

**Conclusions:**

Our results demonstrate an opposed activity of NK cells during retroviral infection. They were protective in the initial phase of infection, when adaptive T cell responses were not yet detectable, but were dispensable for viral immunity after T cell expansion. At later time points they exhibited regulatory functions in inhibiting virus-specific CD8^+^ T cell responses.

## Findings

NK cells are cytotoxic cells of the innate immune system, which contribute to the control of many virus infections. They express a variety of different receptors on their surface driving their functions towards activating or inhibitory responses. NK cells mediate their effector functions through recognition and elimination of virus-infected cells. However, for several viral infections it was shown that NK cells suppress the adaptive immunity by killing virus–specific T cells [1-4]. This resulted in increased viral loads and might contribute to impaired viral clearance. On the other hand it has been demonstrated that HIV uses different mechanisms to escape NK cell responses. HIV modulates the ligand expression of the activating NK cell receptor NKG2D on infected cells resulting in reduced NK cell activation [5]. Additionally, mutational adaptation of HIV has been shown to result in stimulation of inhibitory receptors on NK cells and subsequent escape from NK cell-mediated immune pressure [6]. These opposed mechanisms suggest that NK cells may have distinct effects in different phases of viral infections. Here we analyzed these effects in the murine Friend retrovirus (FV) model by depletion of NK cells at different time points after retroviral infection. The antiviral role of NK cells during an acute FV infection has been investigated in several previous studies. However, almost all of these studies were performed with FV stocks containing Lactate dehydrogenase elevating virus (LDV). Only one recent paper with LDV-free FV showed that NK cells recognize infected target cells by interaction of RAE-1 and NKG2D [7]. LDV induces a massive type I interferon response during acute infection, which we have shown to significantly modulate NK cell responses [8]. Since LDV-free FV induces only barely detectable levels of type I interferons, the previous studies provide very little useful information on the role of NK cells in the immune control of FV infection. We therefore addressed this question in mice that were infected with a LDV-free FV stock.

### Kinetics of FV infection in different lymphoid organs

**Figure 1:**
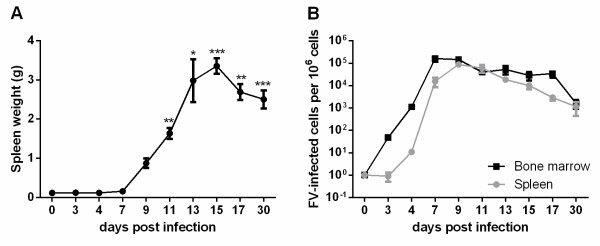
**Kinetics of FV infection.** Spleen weights of FV-infected CB6F1 mice were determined at different time points after FV infection **(A)**. Statistically significant differences compared to naive mice (0 dpi) are indicated by * for p < 0.05; ** for p < 0.01; *** for p < 0.001. Viral loads of FV-infected CB6F1 mice were analyzed in bone marrow and spleen using an infectious center assay **(B)**. At least six mice per group were analyzed and the mean values for each group are indicated by a symbol + SEM.

To characterize the effector functions of NK cells during different phases of FV infection, we first analyzed the kinetics of virus infection in CB6F1 mice, a cross of highly susceptible Balb/c mice and resistant C57BL/6 mice. FV infection resulted in the development of a severe splenomegaly starting at day 9 post infection (dpi) demonstrating their FV susceptibility (Figure 1A). Spleen weights peaked 15 dpi with a significant higher mean weight (3.36 g) compared to naive control mice (0.12 g) and afterwards steadily declined. The kinetics of acute viral loads in spleen and bone marrow of infected mice are shown in Figure 1B. These organs were known to be the main reservoir for FV replication [9]. During initial FV infection, virus-infected cells were detected as early as 3 dpi in the bone marrow and 4 dpi in the spleen, the viral loads peaked 7 and 9 dpi, respectively. Subsequently, the viral loads constantly declined until 30 dpi.

To analyze the impact of NK cells on FV replication, we depleted NK cells during different phases of infection. Here, we focused on three different time points of acute FV infection. 1) The initial phase (3–4 dpi), when only NK cells are able to kill virus-infected cells because T cell responses are not yet developed [9]. 2) At the peak of virus replication (7–15 dpi), when virus-specific T cells are detectable and eliminate infected target cells [9]. 3) The late phase (30 dpi), when viral loads are reduced but FV-induced leukemia starts to develop. Then cytotoxic CD8^+^ T cells become dysfunctional in FV infection [9].

### Anti-retroviral effects of NK cells during initial FV infection

**Figure 2:**
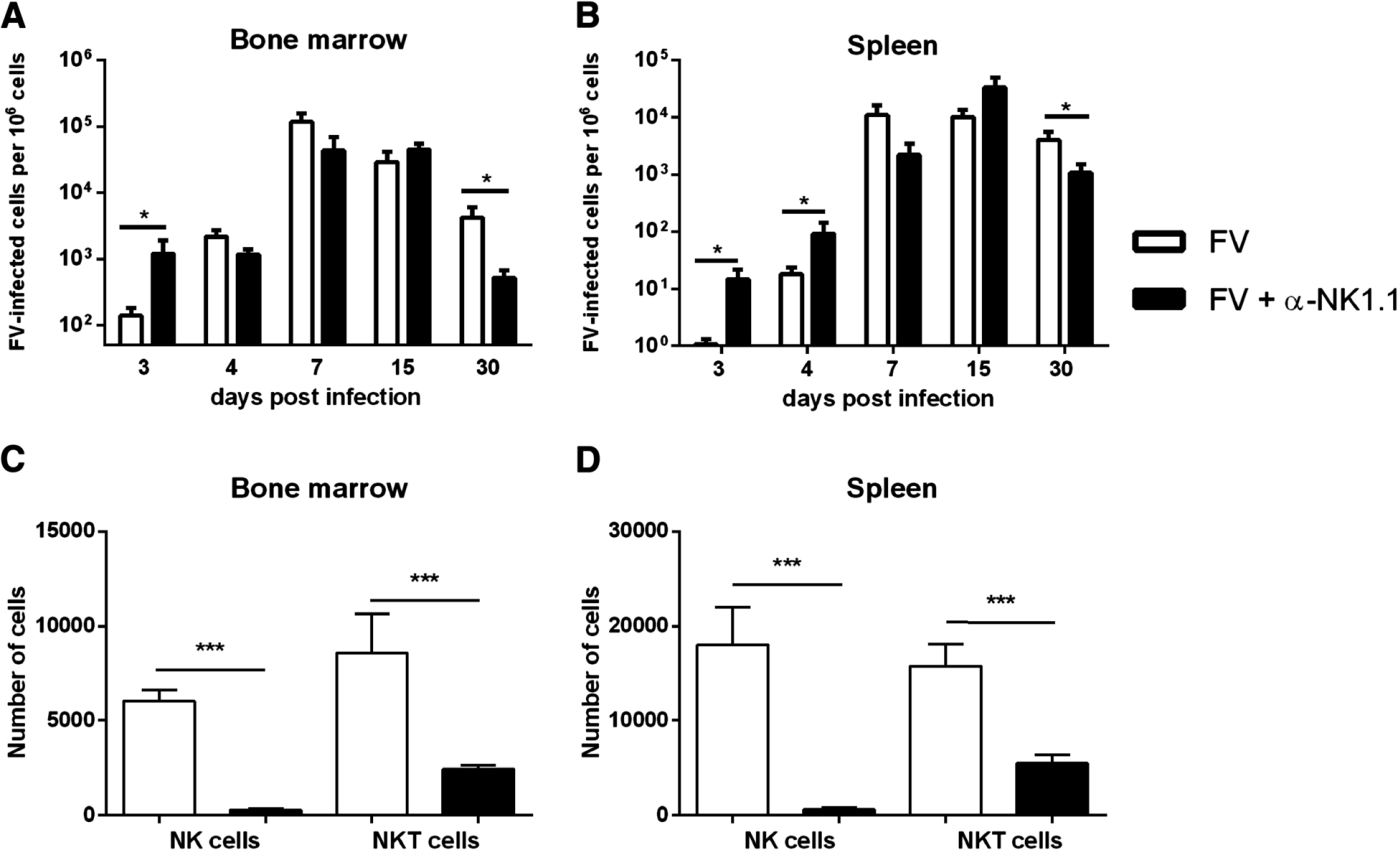
**Viral loads in NK cell depleted FV-infected mice.** Mice were infected with FV and NK cells were depleted by injecting cell culture supernatant containing α-NK1.1 antibody or isotype control (BioXCell). Viral loads were determined in the bone marrow **(A)** and spleen **(B)** by infectious center assay at different phases of infection. A minimum of four mice per group were analyzed and the mean values are indicated by bars + SEM. Experiments were repeated at least twice. Bone marrow cells **(C)** and splenocytes **(D)** were analyzed by flow cytometry in order to distinguish NK cells (CD3^-^ CD49b^+^ NK1.1^+^) and NKT cells (CD3^+^ NK1.1^+^). NK cells were depleted with an efficiency of at least 95%, whereas up to 70% of total NKT cells were ablated. A minimum of seven mice per group were used. Experiments were repeated at least twice. Total numbers of individual cell types per 10^6^ lymphocytes were calculated and indicated by bars + SEM. Statistically significant differences between depleted FV-infected mice and non-depleted FV-infected mice are indicated by * for p < 0.05 and *** for p < 0.001.

**Figure 3:**
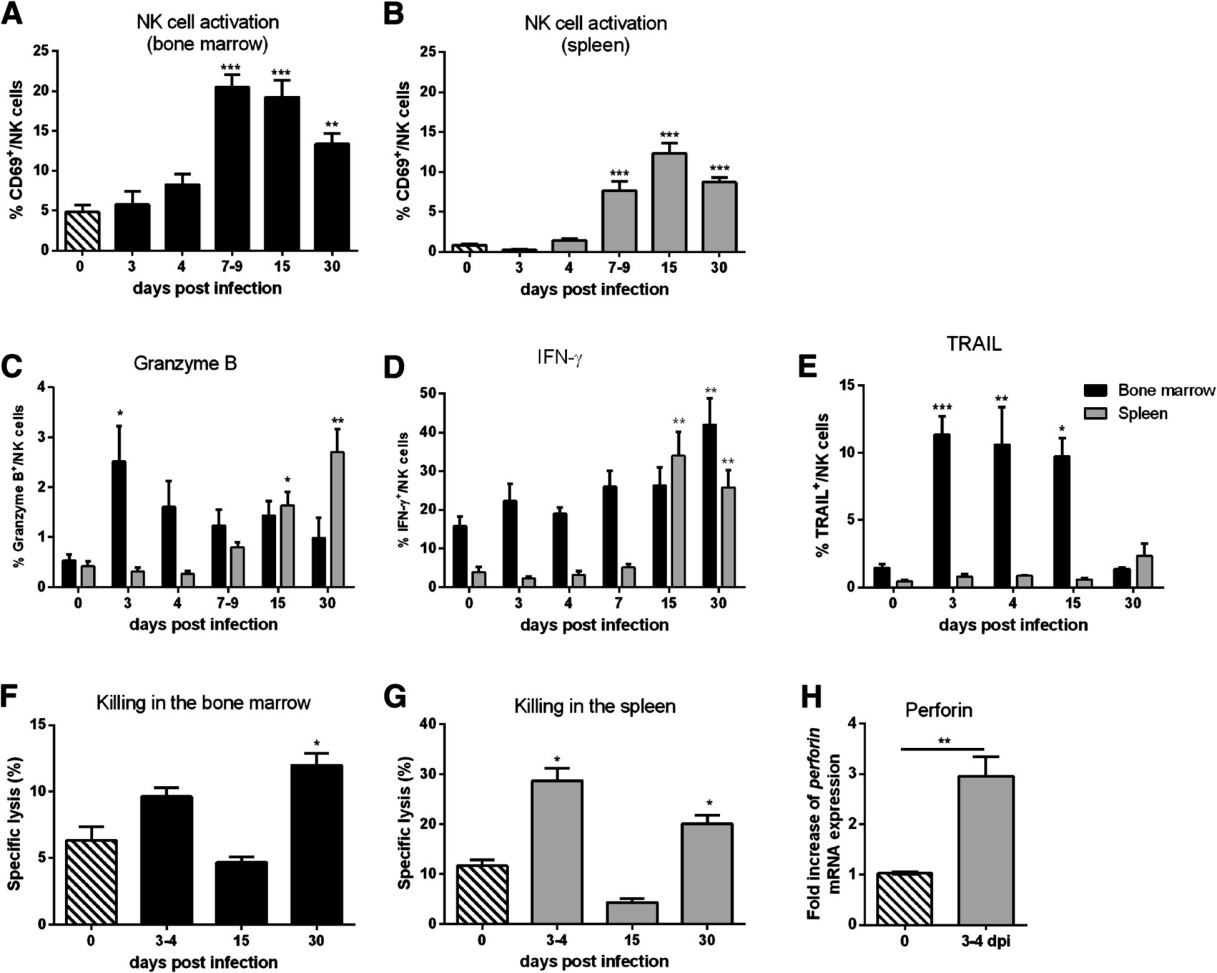
**Activation and effector functions of NK cells.** Bone marrow cells and splenocytes of FV-infected or naive (0 dpi) CB6F1 mice were analyzed by flow cytometry at different time points **(A-E)**. The early activation marker CD69 was used to determine the percentage of activated NK cells (CD3^-^ CD49b^+^ NK1.1^+^ CD69^+^; **A-B**). At least eight mice of minimum two independent experiments were used and the mean values are shown by bars + SEM. The effector functions of NK cells were determined measuring the intracellular expression of granzyme B **(C)**, IFN-γ **(D)** and the surface expression of TRAIL **(E)**. A minimum of seven mice in at least two independent experiments were analyzed. The cytotoxic potential of NK cells in the bone marrow and spleen was analyzed in an *in vitro* NK cell cytotoxicity assay. NK cells were isolated from spleen and bone marrow of FV-infected mice at different time points during infection (3–4 dpi, 15 dpi and 30 dpi) **(F-G)**. MHC-I-negative target cells (YAC-1) were stained with CFSE and co-cultured with isolated NK cells at effector-target ratio of 25:1 for 24 h. At least four mice were used for the analysis. **(H)** Levels of *perforin* mRNA were measured in splenocytes at day 3–4 post FV infection by quantitative real time-PCR. The housekeeping gene β-actin was amplified from each sample to normalize the template concentration and used as an internal standard. At least 2 independent experiments with four mice were performed and the samples were run in duplicate. Statistically significant differences between the groups of infected mice and naive mice are indicated by * for p < 0.05; ** for p < 0.01; *** for p < 0.001.

As *in vivo* depletion with antibodies against NK1.1 has the potential to affect other cell subsets, we analyzed their influence on NKT cells. Figure 2C and 2D illustrate an up to 70% depletion of NKT cells by application of α-NK1.1 in spleen and bone marrow of FV-infected mice. Thus, both cell subsets, NK and NKT cells, might influence anti-retroviral immunity. Figure 2A and 2B indicate that ablation of NK and NKT cells in the initial phase of FV infection (3–4 dpi) resulted in a significant increase in viral loads compared to non-depleted control mice, which was determined by infectious center (IC) assay (for detailed description of experimental procedures, see Additional file 1) and RT-PCR analysis (data not shown). In the bone marrow (Figure 2A) an 8.5-fold increase in viral loads was observed at 3 dpi. In the spleen (Figure 2B) 5-fold to 15-fold elevated viral loads were detected at 4 and 3 dpi, respectively. At this early time point of infection no significant activation of NK and NKT cells, indicated by the expression of CD69, was found (Figure 3A, 3B and data not shown). However, functional activation of NK cells, but not NKT cells (data not shown), were observed, reflected by a significant increase in tumor necrosis factor related apoptosis inducing ligand (TRAIL) and granzyme B expression (Figure 3C and 3E) in the bone marrow of FV-infected mice. In splenic NK cells increased levels of *perforin* mRNA were detected at 4 dpi (Figure 3H) compared to naive controls. The effector function of NK cells during the initial infection was confirmed in an *in vitro* cytotoxicity assay. Increased killing of target cells (YAC-1) was mediated by NK cells from spleen or bone marrow of FV-infected mice (Figure 3F and 3G). Altogether, during the initial phase of FV infection, when T cell responses are not yet developed, NK cells or NKT cells mediate early protective anti-retroviral immunity.

### No effect of NK cells during the peak FV replication

Next, we examined the function of NK cells 7–15 dpi, when T cells expand and control FV replication [9,10]. Depletion of NK and NKT cells during this phase of infection did not influence viral loads in both investigated organs, which was shown by an IC assay (Figure 2A- 2B) and RT-PCR analysis (data not shown). However, at that time point both cell subsets started to express high levels of CD69 (Figure 3A, 3B and data not shown). The expression of the functional molecules granzyme B, interferon-γ (IFN), or TRAIL were elevated only in NK cells from spleen or bone marrow (Figure 3C- 3E), but isolated NK cells failed to eliminate target cells (Figure 3F and 3G) which correlated with the findings from the depletion experiments (Figure 2A- 2B). Thus at 7 to 15 dpi NK cells and NKT cells did not contribute to antiviral immunity, which is mainly mediated by CD8^+^ T cells during peak viral replication [9].

### Negative effects of NK cells during the late phase of acute FV infection

During the late phase of FV infection (30 dpi) susceptible mice start to develop a lethal erythroleukemia. Then virus-infected as well as transformed cells are potential targets for NK cells. To evaluate their role during this phase, we performed depletion experiments starting at 20 dpi. Surprisingly, application of α-NK1.1 antibody resulted in a significant reduction of viral loads at 30 dpi, which was observed by an IC assay (Figure 2A- 2B) and RT-PCR analysis (data not shown). An 8-fold decrease of the viral loads in the bone marrow (Figure 2A) and a 4-fold reduction in the spleen (Figure 2B) was observed compared to control mice. NK cells were still activated (CD69^+^) at this late time point (Figure 3A and 3B) and expressed elevated levels of granzyme B (Figure 3C) and IFN-γ (Figure 3D). TRAIL was not detected on NK cells from mice infected for 30 days (Figure 3E). Next, we analyzed if up-regulation of effector molecules correlated with the cytotoxic potential of NK cells during late acute FV infection. Figure 3F and 3G show that NK cells isolated 30 dpi significantly lysed target cells. 30 dpi NK cells were activated and had an effector phenotype, which allowed them to specifically kill target cells *in vitro*. Surprisingly, NK and NKT cells did not contribute to viral immunity but rather negatively influenced viral loads during the late phase of acute FV infection.

### NK cell depletion improved specific CD8^+^ T cell responses in FV-infected mice

**Figure 4:**
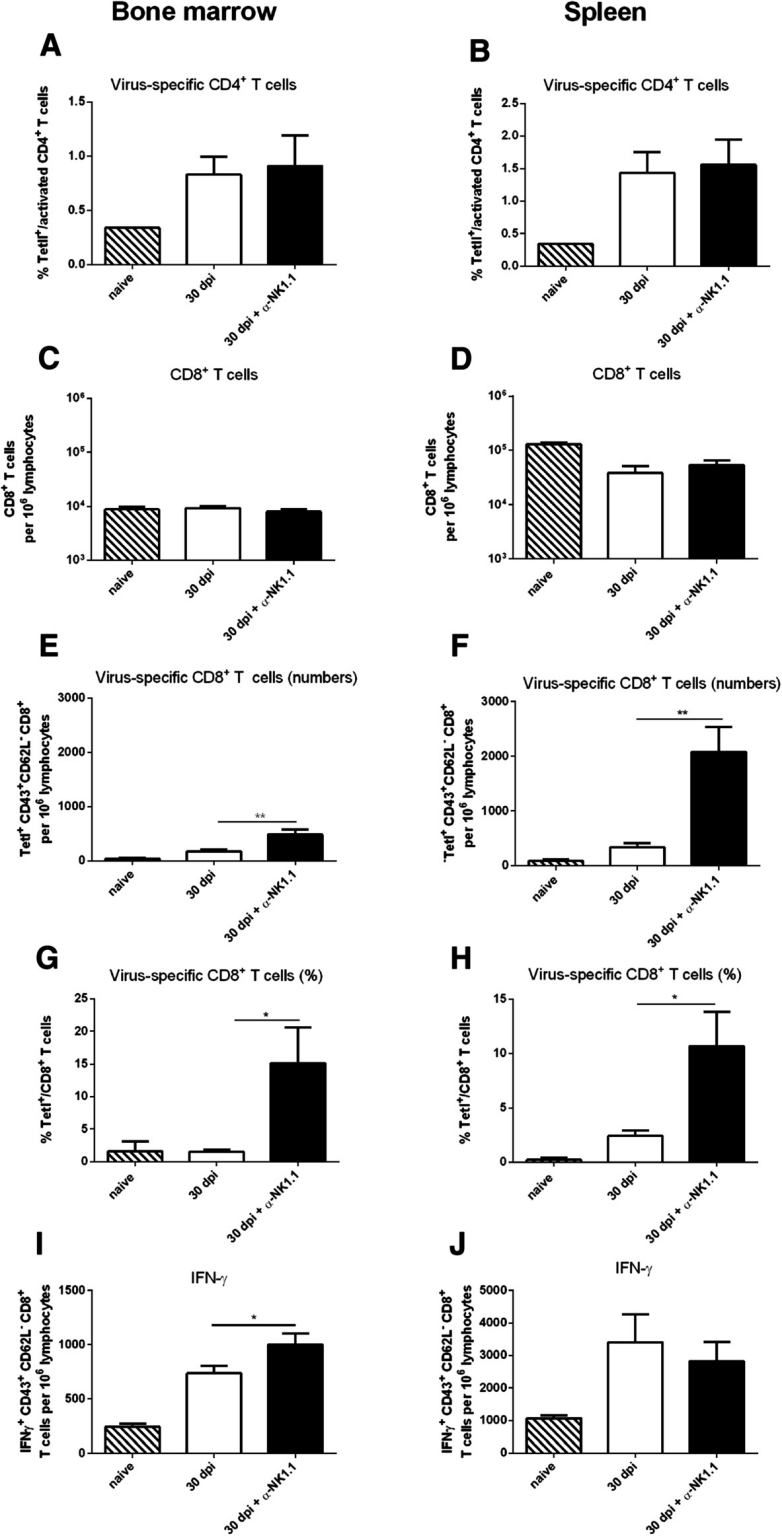
**FV-specific CD4**^**+ **^**and CD8**^**+ **^**T cell responses in the absence of NK cells.** CB6F1 mice were infected with FV and one group of mice was NK cell depleted. Bone marrow and spleen cells were analyzed at 30 dpi. For the analysis of activated (CD43^+^ CD44^+^) virus-specific CD4^+^ T cells, bone marrow **(A)** and splenic **(B)** cells were stained with MHC class II-antibody tetramers specific for F-MuLV env fn20 [11]. Numbers of total CD8^+^ T cells were analyzed by flow cytometry in bone marrow **(C)** and spleen **(D)**. Numbers **(E, F)** and percentages **(G, H)** of activated (CD43^+^ CD62L^-^) effector CD8^+^ T cells, which are specific for the FV gagL epitope, were stained for D^b^gagL class I tetramers and determined by flow cytometry in bone marrow **(E, G)** and spleen **(F, H)**. Bone marrow **(I)** and splenic **(J)** activated CD8^+^ T cells were stained intracellularly for IFN-γ. A minimum of seven mice were used for analysis. At least two independent experiments were performed. Significant differences between the groups were analyzed by using the unpaired Students t test (** for p < 0.01) statistically significant by an unpaired t test (** for p < 0.01).

The depletion experiments revealed that the presence of NK or NKT cells during the late phase of acute FV infection enhanced viral loads. We addressed the question, if these cells impair virus-specific T cell responses. Therefore, mice with or without NK cells and NKT cells were analyzed for their virus-specific T cell responses 30 dpi. Depletion with α-NK1.1 antibody had no significant effect on the percentages of virus-specific CD4^+^ T cells (Figure 4A and 4B). Whereas the depletion significantly increased the frequency and numbers of virus-specific CD8^+^ T cells in both investigated organs without changing the total numbers of CD8^+^ T cells (Figure 4C– 4H). Further analysis of the functionality of effector CD8^+^ T cells revealed a significant higher amount of CD8^+^ T cells producing IFN-γ in the bone marrow of depleted mice (Figure 4I). These data imply that activated NK cells suppress CD8^+^ T cell responses and impair anti-retroviral immunity during the late phase of acute FV infection.

In this report, we analyzed NK cell effector functions and their impact on viral replication and adaptive immune responses during acute retroviral infection. Altogether, we identified an antiviral role of NK cells or NKT cells during the initial FV infection (3–4 dpi), which might contribute to the early control of viral replication. Others could already show that mice deficient in NKT cells were more susceptible in controlling some viruses like HSV-1/2, MCMV, and EMCV (reviewed in [12]). Thus, either NK cells or NKT cells might participate in the early antiviral immunity against FV. Later during infection, CD8^+^ T cells become the key players of anti-retroviral immunity [9] and replace NK and NKT cell responses. However, the strong CD8^+^ T cell responses might have to be counter-regulated in some virus infections to prevent immunopathology. There is growing evidence that NK cells and not NKT cells play a role in this regulation. In the LCMV-model, it was shown that depletion of NK cells alone enhanced T cell responses and thus mice could control viral replication and thereby prevent chronic infection [2]. The NK cell-mediated inhibition of CD8^+^ T cells in this model depended on NKG2D binding and perforin production. Others reported that activated NK cells eliminate CD4^+^ T cells in a perforin-dependent manner, which might indirectly affect CD8^+^ T cell function and exhaustion during LCMV infection [1]. We did not observe an effect of NK cell depletion on CD4^+^ T cell responses suggesting a direct suppressive effect of NK cells on CD8^+^ T cells in our model. Others demonstrated that NK cell depletion during high-dose influenza infection improved the survival rate of infected mice which was not seen in medium- or low-dose influenza infections [3,13,14]. The data indicate that viral loads might predict the effector functions of NK cells. Moreover, during herpes virus infection, a regulatory role of NK cells was described. It was shown that a strong NK cell response can limit T cell responses in MCMV infection [15,16]. Altogether, it seems that the balance of NK and T cell responses seems to be critical for viral immunity and the impact of NK cells depends on the type and dose of virus as well as the phase of virus infection. Thus, it is very important to further investigate the factors that influence NK cell regulatory functions.

## Competing interests

The authors have declared no competing interests.

## Authors’ contributions

EL performed the experiments, analyzed the data and participated in the statistical analysis. SF carried out the experiments. UD conceived and designed the experiments and wrote the paper. KG performed the experiments, analyzed the data, participated in the statistical analysis and wrote the paper. All authors read and approved the final manuscript.

## Supplementary Material

Additional file 1 Material and Method. Click here for file

## References

[B1] Waggoner SN Cornberg M Selin LK Welsh RM Natural killer cells act as rheostats modulating antiviral T cells Nature 2012 481 7381 394 398 22101430 10.1038/nature10624 PMC3539796

[B2] Lang PA Lang KS Xu HC Grusdat M Parish IA Recher M Elford AR Dhanji S Shaabani N Tran CW Natural killer cell activation enhances immune pathology and promotes chronic infection by limiting CD8+ T cellimmunity Proc Natl Acad Sci USA 2012 109 4 1210 1215 10.1073/pnas.1118834109 22167808 PMC3268324

[B3] Zhou G Juang SW Kane KP NK cells exacerbate the pathology of influenza virus infection in mice Eur J Iimmunol 2013 43 4 929 938 10.1002/eji.201242620 23436540

[B4] Soderquest K Walzer T Zafirova B Klavinskis LS Polic B Vivier E Lord GM Martin-Fontecha A Cutting edge: CD8+ T cell priming in the absence of NK cells leads to enhanced memory responses J Iimmunol 2011 186 6 3304 3308 10.4049/jimmunol.1004122 21307295

[B5] Matusali G Tchidjou HK Pontrelli G Bernardi S D’Ettorre G Vullo V Buonomini AR Andreoni M Santoni A Cerboni C Soluble ligands for the NKG2D receptor are released during HIV-1 infection and impair NKG2D expression and cytotoxicity of NK cells FASEB J 2013 27 6 2440 2450 10.1096/fj.12-223057 23395909

[B6] Alter G Heckerman D Schneidewind A Fadda L Kadie CM Carlson JM Oniangue-Ndza C Martin M Li B Khakoo SI HIV-1 adaptation to NK-cell-mediated immune pressure Nature 2011 476 7358 96 100 10.1038/nature10237 21814282 PMC3194000

[B7] Ogawa T Tsuji-Kawahara S Yuasa T Kinoshita S Chikaishi T Takamura S Matsumura H Seya T Saga T Miyazawa M Natural killer cells recognize friend retrovirus-infected erythroid progenitor cells through NKG2D-RAE-1 interactions In Vivo J Virol 2011 85 11 5423 5435 10.1128/JVI.02146-10 21411527 PMC3094956

[B8] Gibbert K Joedicke JJ Meryk A Trilling M Francois S Duppach J Kraft A Lang KS Dittmer U Interferon-alpha subtype 11 activates NK cells and enables control of retroviral infection PLoS Pathog 2012 8 8 e1002868 10.1371/journal.ppat.1002868 22912583 PMC3415439

[B9] Zelinskyy G Dietze KK Husecken YP Schimmer S Nair S Werner T Gibbert K Kershaw O Gruber AD Sparwasser T The regulatory T cell response during acute retroviral infection is locally defined and controls the magnitude and duration of the virus-specific cytotoxic T cell response Blood 2009 114 15 3199 3207 10.1182/blood-2009-03-208736 19671923

[B10] Manzke N Akhmetzyanova I Hasenkrug KJ Trilling M Zelinskyy G Dittmer U CD4+ T cells develop antiretroviral cytotoxic activity in the absence of regulatory T cells and CD8+ T cells J Virol 2013 87 11 6306 6313 10.1128/JVI.00432-13 23536666 PMC3648127

[B11] Shimizu T Uenishi H Teramura Y Iwashiro M Kuribayashi K Tamamura H Fujii N Yamagishi H Fine structure of a virus-encoded helper T cellepitope expressed on FBL-3 tumor cells J Virol 1994 68 12 7704 7708 7525983 10.1128/jvi.68.12.7704-7708.1994 PMC237231

[B12] Diana J Lehuen A NKT cells: friend or foe during viral infections? Eur J Immunol 2009 39 12 3283 3291 10.1002/eji.200939800 19830742

[B13] Gazit R Gruda R Elboim M Arnon TI Katz G Achdout H Hanna J Qimron U Landau G Greenbaum E Lethal influenza infection in the absence of the natural killer cell receptor gene Ncr1 Nat Immunol 2006 7 5 517 523 10.1038/ni1322 16565719

[B14] Stein-Streilein J Guffee J In vivo treatment of mice and hamsters with antibodies to asialo GM1 increases morbidity and mortality to pulmonary influenza infection J Immunol 1986 136 4 1435 1441 3944461

[B15] Andrews DM Estcourt MJ Andoniou CE Wikstrom ME Khong A Voigt V Fleming P Tabarias H Hill GR van der Most RG Innate immunity defines the capacity of antiviral T cells to limit persistent infection J Exp Med 2010 207 6 1333 1343 10.1084/jem.20091193 20513749 PMC2882831

[B16] Lee SH Kim KS Fodil-Cornu N Vidal SM Biron CA Activating receptors promote NK cell expansion for maintenance, IL-10 production, and CD8 T cell regulation during viral infection J Exp Med 2009 206 10 2235 2251 10.1084/jem.20082387 19720840 PMC2757878

