# Supplementary material for: Distinct roles of NK cells in viral immunity during different phases of acute Friend retrovirus infection
Source: Retrovirology. 2013 Nov 1;10:127. doi: 10.1186/1742-4690-10-127 (PMC3826539; doi:10.1186/1742-4690-10-127)
Supplement: Additional file 1 — Material and Method. [file 1742-4690-10-127-S1.docx]

# Additional files

### Additional file 1 – Material and Method

## Mice

Seven to nine weeks old female CB6F1 hybrid mice (BALB/c × C57BL/6 F1; H-2^b/d^ Fv1^b/b^ Fv2^r/s^ Rfv3^r/s^) (Charles River Laboratories, Germany) were used for the experiments. All mice were treated in accordance with the regulations and guidelines of the institutional animal care and use committee of University of Duisburg-Essen.

## Virus and viral infection

The FV stock used in these experiments was FV complex containing B-tropic Friend murine leukemia helper virus and polycythemia-inducing spleen focus-forming virus. The stock was prepared as a 15 % spleen cell homogenate from BALB/c mice infected 14 days previously with 3000 spleen focus-forming units (SFFU). Mice were injected intravenously with 0.1 ml phosphate-buffered saline containing 250 SFFU of FV. The virus stock did not contain lactate dehydrogenase-elevating virus.

## Detection of Friend virus-infected cells in bone marrow and spleen

Infectious centers (IC) were detected by 10-fold dilutions of single-cell suspensions onto *Mus dunnis* cells. Cultures were incubated for 3 days, fixed with ethanol, stained with F‑MuLV envelope-specific monoclonal antibody 720 and developed with peroxidase-conjugated goat anti-mouse antibody and aminoethylcarbazol to detect foci [[1](#_ENREF_1)].

### RNA isolation

Total RNA was isolated from splenocytes utilizing TRIzol reagent (Life Technologies) and Pure Link RNA Micro Kit (Life Technologies). Isolated RNA was dissolved in RNase-free water and stored at −80°C.

### Real time-PCR

Real time-PCR analysis for the quantification of *perforin* mRNA was performed using Power SYBR Green RT-PCR kit (Life Technologies). Primer sequences (Biomers) for tested genes were as follows: *β-actin* 5’-aaatcgtgcgtgacatcaaa-3’, 5’‑caagaaggaaggctggaaaa-3’, *perforin* 5’-gagcccctgcacacattactggaa-3’ and 5’‑acattctcaaagtccatct-3’. The quantitative mRNA levels were performed by using StepOne Software v2.3 (Life Technologies) and were normalized to *β-actin* mRNA expression levels.

**Cell surface and intracellular staining by ﬂow cytometry**

Cell surface staining was performed using the following antibodies: anti-CD3 (17A2, eBioscience), anti-CD49b (DX5, eBioscience), anti-CD69 (H1.2F3, eBioscience), anti-NK1.1 (PK136, BD), anti-TRAIL (N2B2, eBioscience). Dead cells were excluded from analysis (positive for fixable viability dye, eBioscience). For detection of FV-specific T cells bone marrow and spleen cells were stained with anti-CD4 (GK1.5, eBioscience), anti-CD8 (53-6.7, eBioscience), anti-CD43 (1B11, BioLegend), anti-CD44 (IM7, eBioscience) and anti-CD62L (MEL-14, eBioscience). Intracellular IFN-γ (XMG1.2, eBioscience) and granzyme B (clone GB12, BD) staining was performed as described [[2](#_ENREF_2), [3](#_ENREF_3)]. Data were acquired on LSR II flow cytometer (BD) and analyses were performed using FACSDiva (BD) and Flow Jo (Tree Star, USA) software.

## NK cell depletion

Mice were injected intraperitoneally with 0.3 ml supernatant fluid containing NK1.1-specific monoclonal antibody PK136. For depletion of NK cells in the initial phase of FV infection (3-4 dpi), depletion antibody was injected one day prior and one day post FV infection. During the peak of viral replication (7‑15 dpi) we depleted NK cells every other day for at least four times. The last depletion was done one day prior to analysis. In the transition phase of FV infection (30 dpi) we depleted NK cells five times every other day starting at 20 dpi. Control groups received 50 µg of isotype control (mouse IgG2a, BioXCell).

At the days of analysis more than 95 % of NK cells (CD3^-^ CD49b^+^ NK1.1^+^) were depleted in bone marrow and spleen. In addition, up to 70 % of NK T cells (CD3^+^NK1.1^+^) were depleted in FV-infected mice at all analyzed time points. No depletion of γδ or αβ T cells was detected after α-NK1.1 antibody injection. Low dose of α‑NK1.1 antibody (5 µg, BioXCell) still mediated depletion of NK cells (up to 83 %) and NKT cells (up to 70 %) in FV-infected mice.

## Tetramers and tetramer staining

Tetramer staining was performed as described previously [[4](#_ENREF_4)].

## *In vitro* NK cell cytotoxicity assay

*In vitro* NK cell cytotoxicity assay was performed using 1 x 10^4^ CFSE stained YAC-1 tumor cells and 25 x 10^4^ isolated NK cells from spleen or bone marrow of naive or FV-infected mice by MACS technology (Miltenyi Biotec). The cytotoxic assay was performed in 96-well U-bottom plates. The cells were co-incubated for 24 hours in a humidified 5% CO_2_ atmosphere at 37°C. Cells were washed once, resuspended in buffer containing 7-aminoactinomycin D to exclude dead cells and analyzed by flow cytometry.

## Statistical analyses

Statistical analyses and graphical presentations were computed with Graph Pad Prism version 5. Statistical differences between two different groups were determined by the unpaired student’s t test (indicated). Analyses including several groups were tested using Kruskal-Wallis one-way analysis of variance on ranks and Dunn’s multiple comparison test or one-way analysis of variance and Bonferroni multiple comparison test.

# References

1. Dittmer U, Brooks DM, Hasenkrug KJ: **Characterization of a live-attenuated retroviral vaccine demonstrates protection via immune mechanisms.** *J Virol* 1998, **72:**6554-6558.

2. Zelinskyy G, Dietze KK, Husecken YP, Schimmer S, Nair S, Werner T, Gibbert K, Kershaw O, Gruber AD, Sparwasser T, Dittmer U: **The regulatory T-cell response during acute retroviral infection is locally defined and controls the magnitude and duration of the virus-specific cytotoxic T-cell response.** *Blood* 2009, **114:**3199-3207.

3. Zelinskyy G, Kraft AR, Schimmer S, Arndt T, Dittmer U: **Kinetics of CD8+ effector T cell responses and induced CD4+ regulatory T cell responses during Friend retrovirus infection.** *Eur J Immunol* 2006, **36:**2658-2670.

4. Gibbert K, Joedicke JJ, Meryk A, Trilling M, Francois S, Duppach J, Kraft A, Lang KS, Dittmer U: **Interferon-alpha Subtype 11 Activates NK Cells and Enables Control of Retroviral Infection.** *PLoS pathogens* 2012, **8:**e1002868.
